# Experimental evaluation of a self-propelling bio-inspired needle in single- and multi-layered phantoms

**DOI:** 10.1038/s41598-019-56403-0

**Published:** 2019-12-27

**Authors:** M. Scali, P. Breedveld, D. Dodou

**Affiliations:** Delft University of Technology, BioMechanical Engineering, Delft, 2628CD The Netherlands

**Keywords:** Biomedical engineering, Health care

## Abstract

In percutaneous interventions, reaching targets located deep inside the body with minimal tissue damage and patient pain requires the use of long and thin needles. However, when pushed through a solid substrate, a structure with a high aspect ratio is prone to buckle. We developed a series of multi-element needles with a diameter smaller than 1 mm and a length larger than 200 mm, and we experimentally evaluated the performance of a bio-inspired insertion mechanism that prevents needle buckling of such slender structures. The needles consisted of Nitinol wires and advance into a substrate by pushing the wires forward one after the other, followed by pulling all the wires simultaneously backward. The resulting net push force is low, allowing the needles to self-propel through the substrate. We investigated the effect of the needle design parameters (number of wires and their diameter) and substrate characteristics (stiffness and number of layers) on the needle motion. Three needle prototypes (consisting of six 0.25-mm wires, six 0.125-mm wires, and three 0.25-mm wires, respectively) were inserted into single- and multi-layered tissue-mimicking phantoms. The prototypes were able to move forward in all phantoms without buckling. The amount of needle slip with respect to the phantom was used to assess the performance of the prototypes. The six-wire 0.25-mm prototype exhibited the least slip among the three prototypes. Summarizing, we showed that a bio-inspired motion mechanism prevents buckling in very thin (diameter <1 mm), long (length >200 mm) needles, allowing deep insertion into tissue-mimicking phantoms.

## Introduction

**Figure 1:**
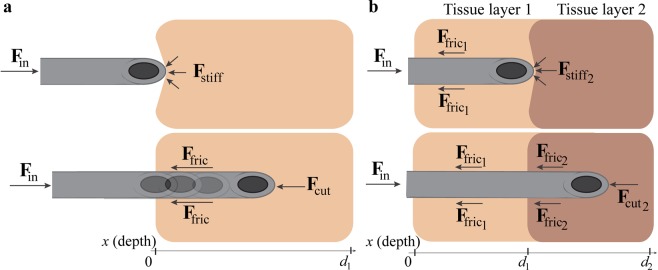
Needle insertion into a body tissue. (**a**) Insertion through a single tissue layer **F**_**in**_ = insertion force, **F**_**stiff**_ = surface stiffness force, **F**_**fric**_ = friction force and **F**_**cut**_ = cutting force. (**b**) Insertion through two tissue layers. ${{\bf{F}}}_{{\rm{f}}{\rm{r}}{\rm{i}}{{\rm{c}}}_{1}}$ is the friction force of tissue layer 1, and ${{\bf{F}}}_{{\rm{s}}{\rm{t}}{\rm{i}}{\rm{f}}{{\rm{f}}}_{2}}$, ${{\bf{F}}}_{{\rm{f}}{\rm{r}}{\rm{i}}{{\rm{c}}}_{2}}$ and ${{\bf{F}}}_{{\rm{c}}{\rm{u}}{{\rm{t}}}_{2}}$ are the surface stiffness force, the friction force, and the cutting force of tissue layer 2, respectively. The axis (*x*) represents the depth of the needle inside the tissue. *x* = 0 is the start of the (first) tissue layer, *x* = *d*_1_ is the end of the (first) tissue layer and the start of the second tissue layer, and *x* = *d*_2_ is the end of the second tissue layer.

Percutaneous procedures, such as biopsy sampling^1,2^, anaesthesia^3,4^, and brachytherapy^5,6^, are minimally invasive interventions where a needle is advanced through soft tissue in order to collect samples, inject drugs, or place radioactive seeds. When a needle is advanced through tissue, forces arise at the needle tip and along the needle body. The force acting on the needle by the surrounding tissue has been described by Okamura *et al*.^7^ as the sum of three components: surface stiffness force (**F**_stiff_), cutting force (**F**_cut_), and friction force (**F**_fric_). In order for the needle to move forward into the tissue, the insertion force (${{\bf{F}}}_{{\rm{in}}}$) applied by the operator has to overcome the sum of these components, that is:

1 $${{\bf{F}}}_{{\rm{in}}}=-\,{{\bf{F}}}_{{\rm{stiff}}}-{{\bf{F}}}_{{\rm{cut}}}-{{\bf{F}}}_{{\rm{fric}}}$$

2 $${{\bf{F}}}_{{\rm{in}}}=\{\begin{array}{c}-{{\bf{F}}}_{{\rm{stiff}}},x=0\\ -{{\bf{F}}}_{{\rm{cut}}}-{{\bf{F}}}_{{\rm{fric}}},0 < x < {d}_{1}\end{array}$$

where *x* is the depth of the needle into the tissue, and *d*_1_ is the end of the tissue layer (Fig. 1a).

The surface stiffness force is due to the elasticity of the tissue and occurs when puncturing the skin or membrane surrounding an organ. The cutting force is generated at the tip of the needle when slicing through the tissue and includes the tissue stiffness force^8^. Assuming that the inner structure of the tissue is homogeneous, the cutting force is independent from the insertion depth. The friction force acts along the length of the needle and is a combination of Coulomb friction, adhesive friction, and viscous friction (i.e., damping)^7^. The Coulomb friction force is linearly dependent on the normal force acting on the needle body as a reaction to the compression applied to the tissue by the needle (also called clamping force^9^). The adhesive friction force is caused by the tendency of the tissue to stick on the needle surface during insertion^10^. The viscous friction force depends on the needle-tissue interaction damping coefficient and the velocity of the needle insertion. In literature, several friction force models for needle-tissue interaction study exist^11^, some of which are simplified, considering only the Coulomb friction force (e.g., Winkler’s foundation model^12^), and others being more complex, also including the adhesive and viscous friction elements (e.g., Karnopp model^7^, Dahl model^13^). Carra *et al*.^13^ presented a model that takes into account the needle passing through different tissue layers, such as skin, fat, muscle, and connective tissue. Each of these layers contributes with its own cutting, stiffness, and friction forces^13,14^. To illustrate, for a substrate consisting of two layers, the insertion force has to overcome the resistance of both layers, that is:

3 $${{\bf{F}}}_{{\rm{in}}}=\{\begin{array}{c}-{{\bf{F}}}_{{{\rm{fric}}}_{1}}-{{\bf{F}}}_{{{\rm{stiff}}}_{2}},x={d}_{1}\\ -{{\bf{F}}}_{{{\rm{fric}}}_{1}}-{{\bf{F}}}_{{{\rm{cut}}}_{2}}-{{\bf{F}}}_{{{\rm{fric}}}_{2}},{d}_{1} < x < {d}_{2}\end{array}$$

where **F**_in_ is the insertion force, ${{\bf{F}}}_{{{\rm{fric}}}_{1}}$ is the friction force within tissue layer 1, ${{\bf{F}}}_{{{\rm{fric}}}_{2}}$ is the friction force within tissue layer 2, ${{\bf{F}}}_{{{\rm{stiff}}}_{2}}$ is the surface stiffness force of tissue layer 2, ${{\bf{F}}}_{{{\rm{cut}}}_{2}}$ is the cutting force of tissue layer 2, and *d*_1_ and *d*_2_ are the positions of the needle when it reaches the end of tissue layer 1 (i.e., the start of tissue layer 2) and the end of tissue layer 2, respectively (Fig. 1b).

When the surface stiffness of the tissue to be penetrated and/or the friction force is high, the **F**_in_ needs to increase to compensate for these forces. If **F**_in_ reaches the critical load of the needle, buckling occurs^15^. Buckling is expressed as a lateral bending of the needle body along its length. Sudden bending of the needle as a result of buckling can damage the surrounding tissue and reduce the accuracy of needle positioning during the procedure^16^.

During puncturing, the critical load (or Euler load) of a slender structure, such as a needle, can be calculated as ${F}_{{\rm{load}}}=\frac{{\pi }^{2}EI}{{(KL)}^{2}}$, where *E* is the Young’s modulus of the needle, *I* is the second moment of area of the cross section of the needle, *K* the effective factor which takes into account the end-condition of the needle (e.g., if both ends are fixed, *K* = 0.5; if both ends are pinned, *K* = 1), and *L* is the unsupported length of the needle. The critical load for a needle being pushed through the tissue is defined by an extended Euler’s load equation ${F}_{{\rm{load}}}=\frac{{\pi }^{2}EI}{{(KL)}^{2}}+\frac{\mu {L}^{2}}{{\pi }^{2}}$, where μ is the spring stiffness of the tissue^17^.

From Euler’s equation, it can be seen that increasing the aspect ratio of the needle decreases the critical buckling load. Long and thin (<1 mm) needles are necessary to be able to reach deep targets in a minimally invasive way. Long and thin needles are, however, by default too flexible to be pushed deeply inside solid structures without buckling, and strategies need to be employed in order to increase the critical buckling load of such needles.

In general, buckling of a slender structure can be prevented by increasing the critical buckling load of the structure or by reducing the axial load applied to the structure^18^. In nature, some species of parasitic wasps are able to insert their slender needle-like structure (aspect ratio up to 260^19^), called ovipositor, without buckling into solid substrates and deposit eggs inside host larvae^19,20^. The ovipositor consists of multiple slender elements, called valves that are interconnected along their length with a tongue-and-groove mechanism and are able to slide along each other. The wasp supports the part of the ovipositor that is outside the substrate with a sheath. The sheath prevents buckling by increasing the cross-section area and by decreasing the unsupported length (see Euler’s equation). For the part of the ovipositor that advances through the solid substrate, the wasp prevents buckling by employing a so-called push-pull mechanism, according to which one valve at the time is pushed forward, while the other valves are pulled backwards. The advantage of this push-pull mechanism compared to pushing of the entire structure at once is that, in the former mechanism, the unsupported length of the valve that is pushed forward (i.e., *L* in Euler’s equation) is decreased, which means that the critical buckling load increases. Additionally, the pushing force of the moving valve is counterbalanced by the pulling force on the other ones, which limits the total axial load applied to the structure (i.e., pushing force), keeping it under the value of the critical buckling load. The presence of directional serrations at the tip allows the valves that are pulled to anchor to the tissue and remain stationary, thereby providing support to the valve that is being pushed^21,22^. As long as the force acting on the stationary valves is higher than the force acting on the pushed valves, the wasp can insert the ovipositor into the substrate while preventing buckling^22^.

Over the past decade, the ovipositor mechanism has inspired researchers to develop novel needle prototypes to overcome buckling during needle insertion^23,24^. Rodriguez y Baena and co-workers presented a series of ovipositor-inspired prototypes with diameters ranging between 12 mm^25^ and 2.5 mm^26^. The probes were made out of four elements, each of them with a bevel-tip and interlocked along their length similar to the wasp ovipositor. One segment at the time was pushed forward while the other three elements were kept stationary. The authors showed that inserting a probe into a substrate using this push-pull motion resulted in less tissue damage than a single-element probe pushed through the tissue^27^.

In previous work^24^, we developed an ovipositor-inspired self-propelling needle with a diameter of 1.2 mm and a length of 160 mm, consisting of seven Nitinol wires (diameter = 0.25 mm) that were connected at the tip and actuated independently. The wires were pushed forward one after the other, while the other wires remained stationary. At the end, by pulling all the wires simultaneously, the needle was able to advance without buckling on a straight path as well as curved paths inside a gelatine phantom.

Currently, thin needles used in clinical practice are short to prevent buckling during insertion, therefore they can only reach superficial targets^28^. Examples of thin, short needles commonly used are needles for vaccinations (22–25 G), which are 16–38 mm long^29^, and for insulin delivery (29–31 G), which are 6–13 mm long^30^. To our knowledge, needles with diameter smaller than 1 mm and length larger than 200 mm (length-to-diameter ratio >200) have not been developed yet. One reason of this might be that increasing the length-to-diameter ratio of the needle increases the risk of buckling (cf. Euler’s load equation: if the diameter decreases, then *I* decreases and so does the critical load *F*_load_), making the insertion of such needle into the tissue very challenging. It is thus relevant to investigate whether the ovipositor-inspired buckling prevention mechanism is functional for ultra-thin long needles.

As mentioned previously, the unsupported length (*L*) has a large impact on the value of the critical buckling load. If the length is decreased by a scale factor 2, the *F*_load_ will increase by a scale factor 4. When using a multiple-element needle with a push-pull mechanism, the diameter of the moving element is smaller than the total needle diameter (e.g., 0.25 mm vs. 1.2 mm), but at the same time the unsupported length is kept very small, by moving the wires forward only for a short distance. The result of this is an increase of the *F*_load_ compared to when the entire needle is pushed forward at once.

Additionally, there is no study that shows how an ovipositor-inspired needle behaves in multi-layered tissue-mimicking phantoms. This type of experiment is useful to understand the needle behaviour in environments that are well-controlled, yet closer than homogeneous phantoms to biological organs, where the needle has to move through several tissue layers with different degrees of stiffness.

In this study, we present three ovipositor-inspired self-propelling needle prototypes with diameters of 0.8, 0.6, and 0.4 mm, and a length of 300 mm. The three prototypes consisted of six Nitinol wires with diameter 0.25 mm, three Nitinol wires with diameter 0.25 mm, and six Nitinol wires with diameter 0.125 mm, respectively. Our aim was to investigate the effect of the substrate properties (i.e., stiffness and number of layers) and the needle design parameters (i.e., number of wires and diameters) on the self-propelling motion. To assess that, the performance of the three needle prototypes was evaluated in single- and multi-layered tissue-mimicking phantoms.

Compared to previous studies, this work presents the following new components: (1) decreasing the diameter of the needle prototype to less than 1 mm and increasing the length to more than 200 mm (i.e., increasing the length-to-diameter ratio to a value > 200), (2) testing a continuous motion, where the elements are pushed and pulled simultaneously to achieve the self-propelling motion, (3) providing a systematic evaluation of various diameters and numbers of wires on substrates of various stiffness degrees in order to gain insight into the functional principles and working envelope of ovipositor-inspired needle systems, and (4) investigating the behaviour of the needle in multi-layer tissue-mimicking phantoms.

## Materials and Methods

### Needle tip design and prototype

Our previous ovipositor-inspired prototypes^24,31^ consisted of seven Nitinol wires connected at the tip with an interlocking ring with seven individual holes through which the wires were fed. One wire was glued to the central hole, whereas the other six wires could slide freely through the six holes arranged in a circle around the central hole. The use of an interlocking ring had two limitations: (1) the use of individual holes left gaps in between the wires, permitting gelatine residues to enter the gaps and push the wires apart, making the trajectory into the substrate less predictable. (2) The presence of the ring at the tip increased the diameter of the needle locally from 0.8 mm (body) to 1.2 mm (tip). In order to solve these two problems, in the prototypes presented here, the interlocking ring was replaced with a thin-walled shrinking tube placed around the wires at the tip and fixated to one of the wires, leaving the rest of the wires free to move back and forth.

Three needle prototypes with diameters of 0.8, 0.6, and 0.4 mm were developed. The first prototype consisted of six Nitinol wires (W) with a diameter (D) of 0.25-mm (called henceforth W6-D0.25), kept together at the tip by a 10-mm long shrinking tube (Vention Medical) with an expanded inner diameter (EID) = 0.813 mm and a wall thickness (W) = 0.013 mm. The second prototype consisted of three Nitinol wires with a diameter of 0.25 mm (W3-D0.25), connected with a 10-mm long shrinking tube with a EID = 0.58 mm and W = 0.006 mm. Finally, the third prototype consisted of six Nitinol wires with a diameter of 0.125 mm (W6-D0.125), connected by a 10-mm long shrinking tube with EID = 0.406 mm and W = 0.006 mm. The wires had a total length of 390 mm, of which 90 mm were placed inside an actuation unit and 300 mm outside.

**Figure 2:**
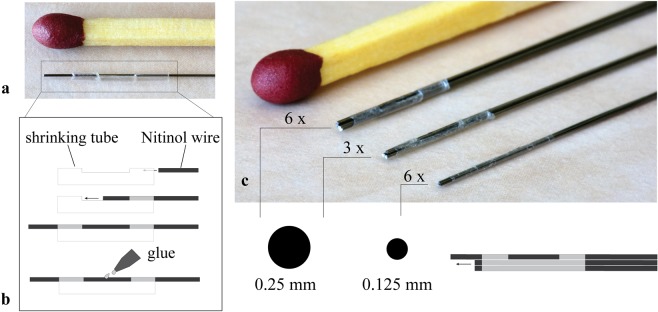
Needle tip design and prototypes. **(a**) Photo of a Nitinol wire connected to a shrinking tube (Vention Medical). (**b**) Stepwise process to fix a Nitinol wire to the tube (**c**) Photos of the tip of the six-wire 0.25-mm prototype (Ø = 0.8 mm), three-wire 0.25-mm prototype (Ø = 0.6 mm), and six-wire 0.125-mm prototype (Ø = 0.4 mm).

The shrinking tube was attached to one of the Nitinol wires in three steps. First, two cuts were made close to each other in the shrinking tube. Second, the wire was passed from the inside of the tube to the outside via the first cut and then re-entered into the tube via the second cut (Fig. 2). Third, the wire was glued (Pattex instant glue, Gold original) to the outside of the shrinking tube in between the two cuts. The other wires were fed through the tube one by one and were able to move freely back and forth relative to the tube.

### Actuation unit

**Figure 3:**
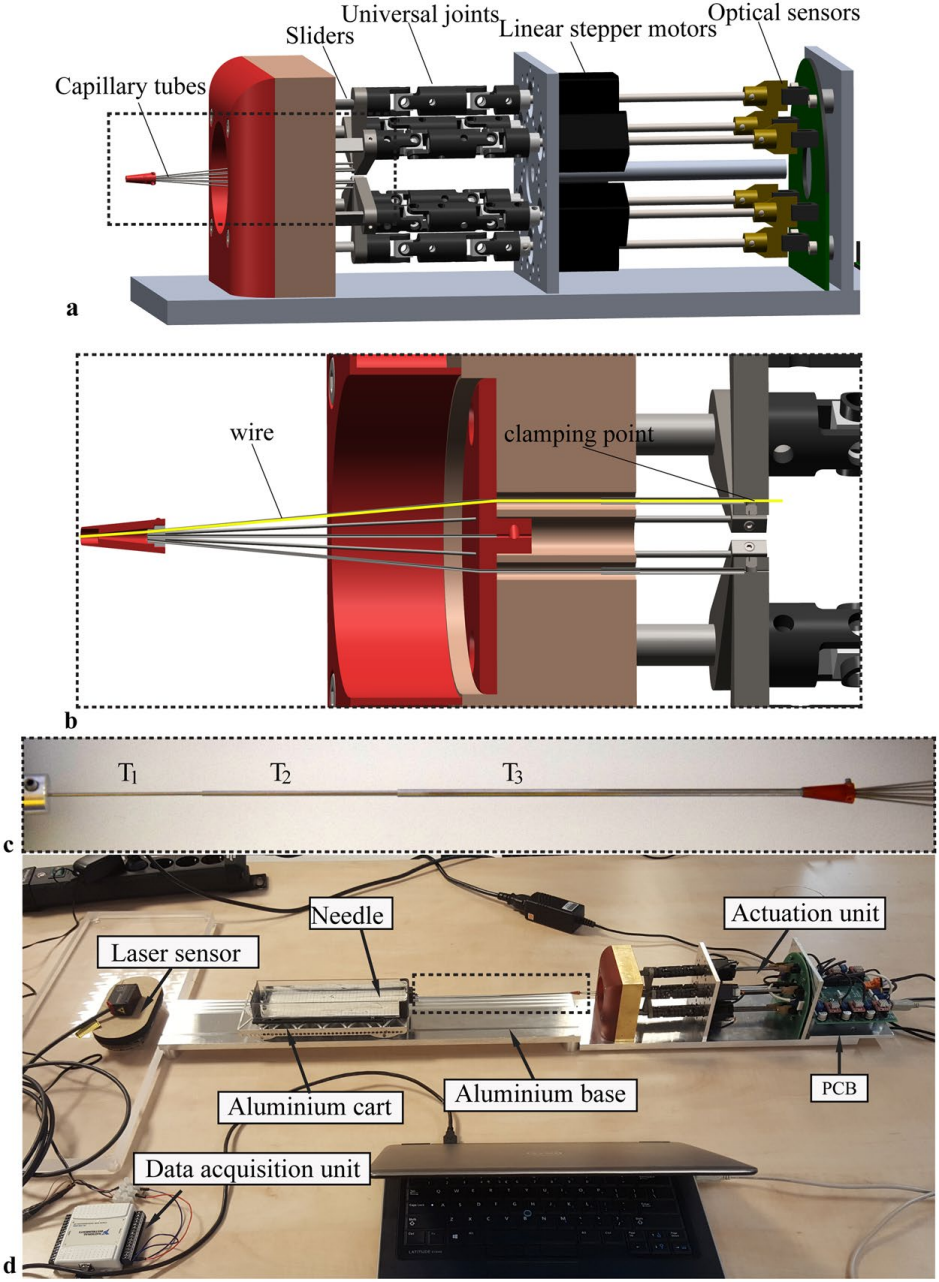
Experimental setup. (**a**) Actuation unit with, from left to right, capillary tubes through which the Nitinol wires are fed, telescopic sliders connected to universal joints, linear stepper motors, and optical sensors (drawing in SolidWorks 2018 by Menno Lageweg/CC by 4.0). (**b**) Section view of the frontal part of the actuation unit showing the clamping point of the wire and the telescopic system serving as a buckling prevention mechanism for the wire (drawing in SolidWorks 2018 by Menno Lageweg/CC by 4.0). (**c**) Telescopic tube system consisted of three capillary tubes (T_1_, T_2_, and T_3_). (**d**) Photo of the experimental setup used during the needle insertion experiments.

The actuation unit (Fig. 3a,b) consisted of six linear stepper motors (RobotDigg Equip Makers) which were able to move each individual wire back and forth. Each wire was clamped to a slider and fed through a telescopic tube system in order to prevent buckling. Bent capillary tubes guided the wires towards the central axis of the system. Universal joints transmitted the motion from the leadscrew of the stepper motors to the sliders. A printed circuit board (PCB) for the control of the stepper motors was developed in-house. The control unit contained an Arduino board (MEGA 2560), stepper motor driver (A4988, Pololu), and LEDs that lit up when the connected motor was running. A set of six optical sensors, placed on a plate behind each of the linear stepper motor, automatically reset the motors to their initial position.

### Experimental setup

The experimental setup (Fig. 3d) consisted of the needle connected to the actuation unit, an aluminium base, an aluminium cart, a gelatine phantom in a customized box, and a data acquisition system. The actuation unit was fixed to the table and aligned to the aluminium base (550 × 100 mm). The aluminium cart (220 × 90 mm) with the box (200 × 50 × 25 mm) was mounted to four ball bearings from which the oil was removed to minimize rolling friction while carrying the gelatine phantom along the aluminium base. A millimetre paper was attached at the bottom of the box and used as reference during needle insertion. Between the actuation unit and the gelatine phantom, the needle was fed through three capillary tubes (T_1_, T_2_, and T_3_) arranged in a telescopic manner to avoid buckling during the experiment (Fig. 3c). Two sets of tubes were used. For the 0.8- and 0.6-mm prototypes, the tube diameters were T_1_ = 2.5 mm, T_2_ = 1.9 mm, and T_3_ = 1.3 mm, and for the 0.4-mm prototype the tube diameters were T_1_ = 1.9 mm, T_2_ = 1.3 mm, and T_3_ = 0.9 mm. The lengths of the tubes were T_1_ = 10 mm, T_2_ = 10 mm, and T_3_ = 11 mm. A laser proximity sensor (MicroEpsilon optoNCDT ILD1420–200, range: 200 mm) was used to record the position of the cart during the experiments. A data acquisition unit (NI USB-6211, 16-bit) in conjunction with LabVIEW 2013 was used to collect the laser sensor data at a sampling frequency of 5 Hz.

### Actuation mode

The wires were actuated following two actuation modes. In the first actuation mode, called *step-by step motion*, the wires were moved forward one by one over a pre-defined distance, called *stroke*. Once all the wires reached that distance, they were simultaneously pulled backwards over the same stroke. We will call henceforth each sequence of a forward and backward motion a *cycle*. In the second phase of each cycle, when the wires had all been pulled backwards, the needle pulled itself forward inside the substrate at a distance equal to the stroke.

### Gelatine phantoms

Gelatine powder (Dr. Oetker Professional, the Netherlands) was mixed with heated tap water (ca. 100 °C) to create gelatine phantoms. For the experiments, 5% and 10% weight of gelatine powder in water (wt) was used. These concentrations correspond to Young’s moduli of, respectively, 5.3 kPa and 17 kPa, measured using a AR-G2 rheometer with parallel plates of 25 mm in diameter as in Scali *et al*.^32^ (see Supplementary Methods). Soft tissues in the human body exhibit a wide range of stiffness degrees, from 0.1 kPa for the brain to 100 kPa for soft cartilage^33^. Indicatively, the 5% wt gelatine approximates healthy liver tissue (<6 kPa), and the 10% wt gelatine approximates cirrhotic liver (>12 kPa)^34^.

Customized containers with 190 × 47 × 30 mm boxes were used for the liquid mixture of gelatine and water. The layers of the gelatine phantoms were created in two steps. First, 5% and 10% wt gelatine phantoms were prepared separately and stored overnight at 4 °C. Then, they were cut in half and the 5% wt layer was placed next to the 10% wt layer. To ensure that the layers are attached, the cut surface of each part was covered with a layer of liquid mixture of 5% wt before putting the layers back into the box. Finally, extra liquid mixture of 5% wt was poured to fill any gap on the contact surface between the two layers. After this process, the boxes were stored for 2 hours at 4 °C in order to solidify the connective part between the gelatine layers.

In total, three gelatine phantom types were used: (1) single-layered phantom consisting of 5% wt gelatine (called henceforth G5) with a total length of 190 mm; (2) multi-layered phantom consisting of one layer 5% wt and a second layer 10% wt gelatine (G5-10), each layer being 95 mm long; and (3) multi-layered phantom consisting of two layers 5% wt separated by a 10-mm layer of 10% wt gelatine (G5-10-5), the first layer 5% wt being 95 mm long and the second 85 mm.

### Self-propelling motion

**Figure 4:**
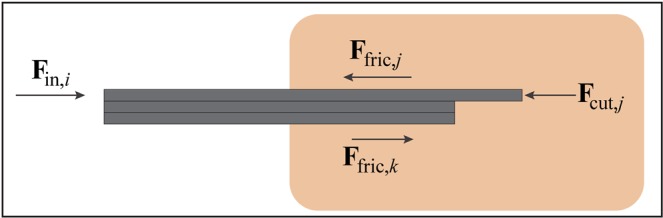
Bio-inspired needle insertion into a substrate. **F**_**in**,***i***_ = total insertion force acting on the needle. **F**_**fric**.***j***_ = friction force on the protruding wires. **F**_**cut**,***j***_ = cutting force acting at the tip of the protruding wires. **F**_**fric**,***k***_ = friction force on the non-protruding wires.

In the case of a needle is made out of multiple wires, Eq. (2) becomes

4 $$\mathop{\sum }\limits_{i=1}^{N}{{\bf{F}}}_{{\rm{in}},i}=-\mathop{\sum }\limits_{i=1}^{N}({{\bf{F}}}_{{\rm{fric}},i}+{{\bf{F}}}_{{\rm{cut}},i})=-\mathop{\sum }\limits_{j=1}^{p}({{\bf{F}}}_{{\rm{fric}},j}+{{\bf{F}}}_{{\rm{cut}},j})-\mathop{\sum }\limits_{k=1}^{np}{{\bf{F}}}_{{\rm{fric}},k}$$

where *N* is the total number of wires, *p* is the number of protruding wires, and *np* is the number of non-protruding wires, assuming that no cutting force acts on the non-protruding wires ($\mathop{\sum }\limits_{j=1}^{np}{{\bf{F}}}_{{\bf{cut}},j}=0$) (Fig. 4).

As shown in previous works^24,35^, the needle is able to propel forward through the substrate with zero net insertion force ($\mathop{\sum }\limits_{i=1}^{N}{{\bf{F}}}_{{\rm{in}},i}=0$) or even net pulling force ($\mathop{\sum }\limits_{i=1}^{N}{{\bf{F}}}_{{\rm{in}},i} < 0$) if

5 $$-\mathop{\sum }\limits_{j=1}^{p}({{\bf{F}}}_{{\rm{fric}},j}+{{\bf{F}}}_{{\rm{cut}},j})-\mathop{\sum }\limits_{k=1}^{np}{{\bf{F}}}_{{\rm{fric}},k}\le 0$$

To achieve this, the sum of the friction force and the cutting force of the protruding wires should be smaller than the friction force acting on the non-protruding wires.

6 $$-\mathop{\sum }\limits_{j=1}^{p}({{\bf{F}}}_{{\rm{fric}},j}+{{\bf{F}}}_{{\rm{cut}},j})\le \mathop{\sum }\limits_{k=1}^{np}{{\bf{F}}}_{{\rm{fric}},k}$$

Once the tissue has been cut (i.e., rupture event), the value of cutting force decreases while the friction force increases with the insertion depth^36,37^. Equation (6) can be satisfied by keeping the number of protruding wires smaller than the number of non-protruding wires (p < np), so that the difference between the forces acting on the two groups of wires increases. In the same way, if the contact area between the protruding wires and the substrate is smaller than that of the stationary wires, the friction force on the body of the protruding wires will be lower than the force on the stationary wires. At a certain depth, an equilibrium between the forces acting on the stationary and protruding wires is reached, $\mathop{\sum }\limits_{j=1}^{p}({{\bf{F}}}_{{\rm{fric}},j}+{{\bf{F}}}_{{\rm{cut}},j})=\mathop{\sum }\limits_{k=1}^{np}{{\bf{F}}}_{{\rm{fric}},k}$. After that, needle advancement with low net push force is possible, that is, the needle can self-propel through the substrate.

### Hypotheses

The slip of the needle per cycle with respect to the gelatine and the number of cycles needed for the needle to travel until a predefined depth were used to assess the effectiveness of the self-propelling of the needle. Assuming that all wires have the same tribological characteristics, that the tips of all wires are identical, that there is perfect contact between the wire and the gelatine, and that the cutting force is negligible, the following hypotheses with respect to the slip of the needle during forward motion through the gelatine were investigated:

1. For a constant number of wires, slip is independent from the wire diameter, because the self-propelling motion relies on the relative friction between the protruding and non-protruding wires.
2. For a constant wire diameter, slip decreases with an increasing number of wires, because the difference between non-protruding and protruding wires is larger in the former case than in the latter.
3. Slip is constant throughout a substrate with constant stiffness.
4. Slip increases when the needle transits from a softer to a stiffer substrate, because the needle encounters more resistance to cut through the stiffer substrate.

### Experimental procedure

The procedure for each measurement was as follows:

1. A gelatine phantom (mean ± standard deviation = 170 ± 0.6 g) was placed inside the box on the cart.
2. The smallest tube of the telescopic system was inserted manually through a hole at the front side of the box for 40 mm inside the gelatine, to ensure that the prototype was inserted in a straight direction. After that, the tube was retracted by 30 mm and fixed at this position so that it does not move during the experiments.
3. The laser sensor was turned on and the needle actuation started. Every measurement was performed with a speed of 2 mm/s and a stroke of 4 mm.
4. The actuation of the needle was let run for 30 cycles or stopped manually once the needle reached an insertion depth of 85 mm.

The needle was cleaned with water after each measurement. A new gelatine phantom was used for each measurement, except when the measurement had to be repeated because of mispositioning of the needle or of the cart. To avoid systematic errors due to the assembly and disassembly of the needles in the actuation unit, the three prototypes were tested one after the other. First the W6-D0.25 was tested in G5, G5-10, and G5-10-5, then the W6-D0.125 was tested in the same three gelatine phantoms, and finally the W3-D0.25 was tested in G5-10. Each prototype-gelatine phantom combination was tested ten times in a randomized order.

During the experiments with the six-wire 0.125-mm prototype, we noticed that, after a certain depth, one or more wires started to buckle in about 50% of the measurements, in particular when the needle was puncturing the 10% layer or was inside the 10% layer. When this happened, the test was stopped and the measurement was repeated.

### Data analysis

The raw data recorded by the laser sensor during the tests were imported in MATLAB 2016b. The data included the time and the position of the cart during the test. A Savitzky-Golay filter was used for smoothing the data. The local maxima peaks in the plot represent the position of the cart after pulling backwards the six wires, and the local minima valleys depict the position of the cart after the six wires have been pushed forward one by one. The distance between two peaks defines a cycle. The peaks and the valleys were detected by applying the function *findpeaks* to the data. We calculated the slip ratio (*S*_ratio_) per each cycle as

7 $${S}_{{\rm{ratio}}}=1-\frac{{d}_{{\rm{meas}}}}{{d}_{{\rm{theor}}}}$$

where *d*_meas_ is the measured distance (i.e., difference between two peaks) and *d*_theor_ is the theoretical distance travelled (i.e., 4 mm per cycle).

We evaluated the number of cycles at three depths, 55 mm (*d*_1_), 65 mm (*d*_2_), and 85 mm (*d*_3_), where *d*_1_ is the depth at which the needle reaches the 10% wt layer in the phantoms G5-10 and G5-10-5, *d*_2_ is the depth at which the needle transits from the 10% wt layer to the 5% wt layer in G5-10-5, and *d*_3_ is the final depth chosen for all measurements. The number of cycles needed to reach each of the three depths was calculated per phantom. Theoretically, with no slip, the needle should reach *d*_1_ in 14 cycles, *d*_2_ in 16 cycles, and *d*_3_ in 21 cycles. Quantitative data were expressed as mean ± standard deviation (*SD*).

### Statistical analysis

We investigated the performance of the three prototypes during three ranges of cycles, each consisting of 5 cycles: the first 5 cycles (Range 1), from the depth of 55 mm up to the 55 mm + 5 cycles (Range 2), and the 5 cycles preceding the final depth of 85 mm (Range 3). These ranges represent the start, middle, and end of the needle motion inside the gelatine phantom. Independent two-tailed *t*-tests were used to compare the two prototypes with different wire diameter (W6-D0.25 vs. W6-D0.125) and the two prototypes with different number of wires (W6-0.25 vs. W3-0.25) at each of the three ranges. One-way ANOVA and Tukey’s post-hoc tests were used to compare the slip ratio between the three gelatine phantoms for the W6-D0.25 prototype and the W6-D0.125 prototype. We conducted one ANOVA test per range (Range 1, Range 2, and Range 3), which led to a total of six statistical comparisons. The significance level was set at *p* < 0.001. The analysis was done in MATLAB 2016b.

## Results

### Six-wire 0.25-mm prototype, step-by-step motion, three gelatine phantoms (5%, 5–10%, 5-10-5%)

**Figure 5:**
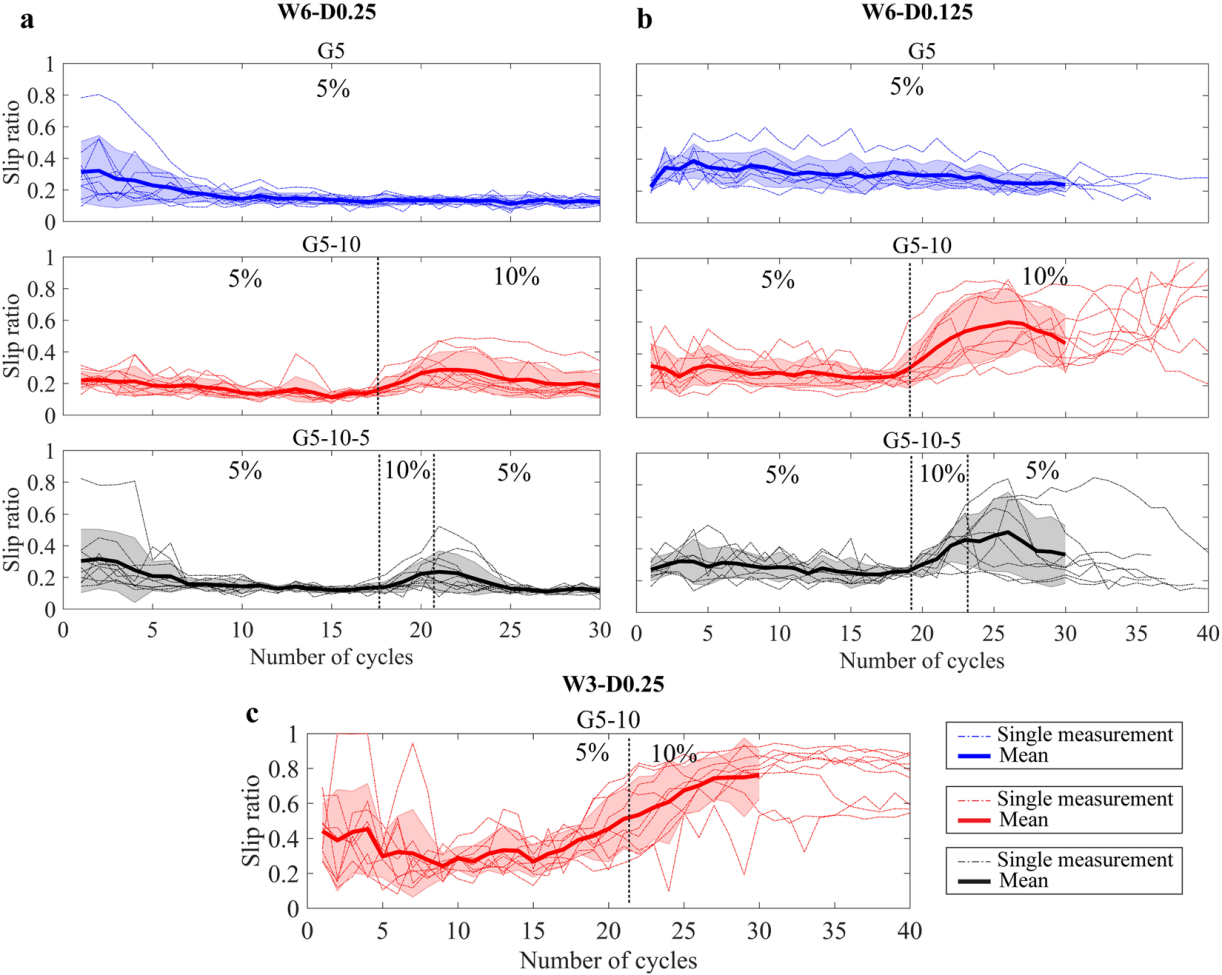
Slip ratio per number of cycles during the experiments into phantoms 5% wt (G5), 5–10% wt (G5-10), and 5%-10%-5% (G5-10-5). (**a**) Six-wire 0.25-mm prototype (W6-D0.25) actuated with step-by-step motion. (**b**) Six-wire 0.125-mm (W6-D0.125) prototype actuated with step-by-step motion. (**c**) Three-wire 0.125-mm (W3-D0.25) prototype actuated with step-by-step motion. The dashed thin lines represent the single measurements, the thick line is the mean value. The area around the mean value represents the standard deviation. The dashed vertical lines indicate the mean number of cycles for the prototypes to reach the different gelatine layers.

Figure 5a shows the slip ratio over the number of cycles for each gelatine phantom. The mean slip ratio when the needle moved through 5% wt gelatine was about 0.2 and increased to 0.3 once the needle entered the 10% wt layer (in G5-10 and G5-10-5). In G5-10-5, the slip ratio returned to the value of 0.2 once the needle transited from the 10% to the 5% wt layer. The variability of the slip ratio was lower in 5% wt than in 10% wt.

Between cycles 1 and 5, the slip ratio was higher in G5 and G5-10-5 than G5-10. For cycle 6 and higher, the slip ratio remained relatively constant. The depth of 55 mm was reached after 17 cycles in 60% of the measurements in G5 and in 50% of the measurements in G5-10 and G5-10-5. After 20 cycles, the second depth (65 mm) was reached in 50% of the measurements in G5 and in 40% of the measurements in G5-10 and G5-10-5. The final depth (85 mm) was reached after 25 cycles in 40% of the measurements in G5 and G5-10-5, and in 20% of the measurements in G5-10 (see Supplementary Fig. S1).

### Six-wire 0.125-mm prototype, step-by-step motion, three gelatine phantoms (5%, 5–10%, 5-10-5%)

We excluded two measurements in each of G5 and G5-10-5 and one measurement in G5-10, because of sensor data failure or failure in reaching the final reference distance of 85 mm. The final number of measurements included in the analysis is eight for G5, nine for G5-10, and eight for G5-10-5.

The mean slip ratio when the needle moved through 5% wt gelatine was about 0.3 and increased to 0.5 once the needle entered the 10% wt layer (in G5-10 and G5-10-5). The variability of the slip ratio was lower in 5% wt than in 10% wt. In G5-10-5, the slip ratio decreased again once the 10% wt layer was passed (Fig. 5b).

The depth of 55 mm was reached after 17 cycles by one measurement in G5-10 and G5-10-5, while the majority (90%) of the measurements needed between 19 and 21 cycles. After 24 cycles, the second depth (65 mm) was reached in 75% of the measurements in G5, in 65% of the measurements in G5-10, and in 50% of the measurements in G5-10-5. The final depth (85 mm) was reached after 30 cycles in 89% of the measurements in G5-10 (see Supplementary Fig. S2).

### Three-wire 0.25-mm prototype, step-by-step motion, one gelatine phantom (5–10%)

One out of the ten measurements was excluded, because of incorrect data registered by the sensor. The mean slip ratio was approximately 0.3 during the motion of the needle inside 5% wt layer (Fig. 5c). At the entrance of 10% wt, the median slip ratio increased up to 0.8. Between cycles 1 and 5, the value of the mean slip ratio was higher than in between cycles 5–15 (0.4 vs 0.2). The variability of the slip ratio was lower in 5% wt than in 10%wt.

The depth 55 mm was reached within 20 cycles in 44% of the measurements, the rest of measurements needed between 20 and 25 cycles. Within 30 cycles, the second depth (65 mm) was reached in 55% of the measurements. The final depth (85 mm) was reached in more than 50 cycles in 66% of the measurements (see Supplementary Fig. S3).

### Statistical analysis

**Figure 6:**
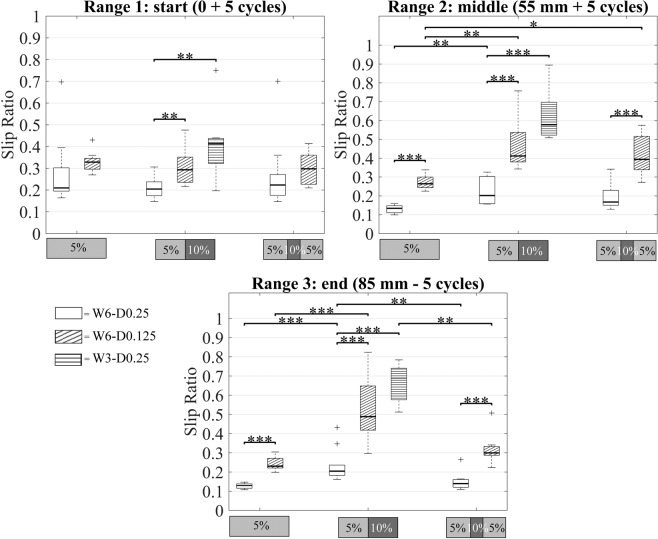
Slip ratio in Range 1, Range 2, and Range 3 for G5 (5%), G5-10 (5–10%), and G5-10-5 (5%-10%-5%). W6-D0.25 (no hatches), W6-D0.125 (diagonal hatches), and W3-D0.25 (horizontal hatches) prototypes. ***p < 0.001, **p < 0.01, *p < 0.05.

The slip ratio was significantly lower for the prototypes with the larger wire diameter in Ranges 2 and 3 for each gelatine phantom. The slip ratio was also significantly lower for the prototypes with the larger number of wires (i.e., six wires) in Ranges 2 and 3 (see Table 1 and Fig. 6 for the complete analysis).

**Table 1:** Results of the independent two-tailed *t*-tests. *t*- and *p*-values, and the corresponding degrees of freedom (*df*), are reported for each range of distance within each gelatine phantom.

Prototypes	Gelatine phantom	Range	*t*-value	*df*	p-value
W6-D0.25 vs. W6-D0.125	G5	1	−0.857	16	0.404
		2	−9.911	16	**<0.001**
		3	−9.518	16	**<0.001**
	G5-10	1	−3.010	17	0.008
		2	−5.057	17	**<0.001**
		3	−4.795	17	**<0.001**
	G5-10-5	1	−0.393	16	0.6993
		2	−5.483	16	**<0.001**
		3	−5.680	16	**<0.001**
W6-D0.25 vs. W3-D0.25	G5-10	1	−3.605	17	0.002
		2	−8.520	17	**<0.001**
		3	−9.411	17	**<0.001**

*Note*. A negative *t*-value indicates that the mean value of the first prototype is smaller than the second. *p* < 0.001 is annotated in bold.

A significant difference was found between the slip ratio values in the three gelatine phantoms in Range 3 for the six-wire 0.25-mm prototypes, *F*(2,27) = 9.77, *p* < 0.001, and the six-wire 0.125-mm prototype, *F*(2,22) = 14.14, *p* < 0.001. The Tukey’s post-hoc test revealed that the slip ratio in 5% wt was significantly lower than the slip ratio in 5–10% wt (*p* < 0.001) for both prototypes.

### Additional experiments

We performed a series of additional experiments to investigate the needle behaviour inside tissue-mimicking phantoms:

1. We used a second actuation mode, called *continuous motion*, where all wires were actuated simultaneously and were always in motion: one wire moved forward while at the same time the other wires moved backward. When a wire reached the stroke value, it started to move backward while the second wire started to move forward and so on, thus creating a continuous needle motion. We performed ten measurements with the W6-D0.25 prototype inside a G5-10 multi-layered phantom (see Supplementary Fig. S4). The needle reached the reference depth of 55 mm in on average 266 s (*SD* = 26 s), 65 mm in 322 s (*SD* = 27 s), and 85 mm in 437 s (*SD* = 40 s). Considering that the motion is continuous, that is, there are no cycles as in the step-by-step motion, the slip ratio was calculated for the total motion (i.e., from the initial point to the final depth). The slip ratio was 0.28 at 55 mm depth, 0.29 at 65 mm depth, and 0.32 at 85 mm depth.
2. We used 15% wt gelatine phantoms (corresponding to 31 kPa) to assess the performance of the needle inside stiff tissues. We performed two tests with the six-wire 0.25-mm prototype actuated with step-by-step motion (speed = 2 mm/s, stroke = 4 mm). The results (see Supplementary Fig. S5) showed that the slip ratio increased from 0.2 to 0.8 when the needle moved from the 5% to the 15% layer. When the needle moved further inside the 15% layer (ca. 10 cycles), the slip ratio decreased, reaching a constant stable value.
3. We investigated the behaviour of the needle inside a 5% wt gelatine phantom with a thin layer of plastic foil at the depth of 95 mm. The foil was used to mimic a serous membrane. The needle was able to penetrate the plastic foil in about 5 cycles (Fig. 7a,b). After puncturing the plastic foil, the needle was able to move through the gelatine with a constant slip ratio of about 0.2 (see Supplementary Video S1).

**Figure 7:**
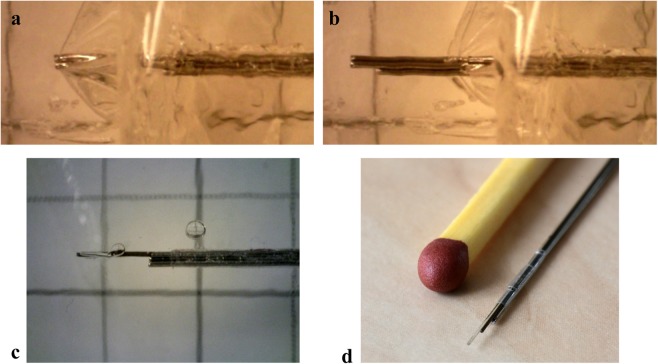
Six-wire 0.25-mm needle prototype moving through a plastic foil placed between two layers of 5% gelatine. **(a**) The needle is pushing the plastic foil without cutting it. (**b**) The needle cuts the plastic foil and moves forward. Photos made with a Digitale PC-Microscoop Dino-Lite (AM73915MZTL 5 Megapixel, 10–140x). (**c**) Injection of water into a 5% wt phantom with the W6-D0.25 prototype where a Nitinol wires is replaced by a Nitinol tube. (**d**) Tip of the W6-D0.25 prototype with two Nitinol wires replaced by a Nitinol tube and an optical fibre.
4. We performed a qualitative test to show the application potential and functionalization of the needle prototype. We replaced one Nitinol wire with a Nitinol tube (Ø = 0.2 mm) and we connected it to a syringe. Then, we manually inserted the needle into a gelatine phantom and push liquid through the Nitinol tube using the syringe. We could inject water inside the gelatine in the area around the needle tip, which indicates that the needle could be used as injection tool (Fig. 7c). Additionally, we replaced one Nitinol wire (Ø = 0.25 mm) with an acrylate recoated optical fibre (Ø = 0.2 mm). Then, we actuated the needle using the step-by-step sequence. The measurement showed that the needle was still able to propel itself forward and the optical fibre could move through the gelatine phantom as well. The used fibre had a smaller diameter than the Nitinol wire, which means that there was a slightly smaller surface in contact with the substrate. The optical fibre could be also placed in the centre of the needle with the six Nitinol wires around it forming a circle (Fig. 7d).

## Discussion

In this work, we investigated the influence of the substrate properties (stiffness) and the needle design parameters (number of wires, outer needle diameter) on the self-propelling motion of a bio-inspired needle through tissue-mimicking phantoms. The results of the experiments showed that the slip ratio is significantly lower for a larger diameter (0.25 vs. 0.125 mm) and for a greater number of wires (six vs. three). Hypothesis 1 is not confirmed, as W6-D0.25 exhibited lower slip than W6-D0.125, which means that the difference between the forces acting on the protruding and non-protruding wires in W6-D0.25 was higher than in W6-D0.125. This might have happened because, during the incremental motion, the wires crack the gelatine in such a way that the contact between gelatine and the wire surface is partially lost for the thinner wires. Additionally, the friction of the ball bearing system used for the cart, albeit low, might play a larger role against the forward motion of the needle into the gelatine when using 0.125 mm compared to 0.25 mm. Moreover, at a length of 300 mm, the 0.125 mm wires are more flexible than 0.25 mm; (limited) buckling along the length (e.g., outside the gelatine) might occur more easily for the thinner wires. Summarizing, the self-propelling mechanism is theoretically size-independent because it relies to relative friction differences, but in reality, side-effects related to the experimental setup have been likely at place, for which reason the results were not supportive to the first hypothesis. The results provided support to Hypothesis 2. The slip ratio was constant through a gelatine layer with the same stiffness, providing support to Hypothesis 3, with a visible increase of the value at the passage from 5% to 10% layer, consistent with Hypothesis 4. This is due to the fact that the needle had to puncture the new layer (10%) which is stiffer than the previous one (5%). When the needle passed from the 10% layer to the 5% layer, the slip ratio decreased. In this case, the needle does not encounter a high stiffness force and can easily penetrate the new layer. The peaks of the value of the slip ratio in Fig. 6 are also a representation of what happens during the insertion of the needle into multi-layered tissue. Prior the puncture, the tissue deforms and the insertion force reaches a peak equal to the stiffness of the tissue. When the tissue layer is punctured, the insertion force value drops and the needle can penetrate the tissue^12,13^. So, the change of slip during the needle insertion is an indication of the needle being in between two layers.

In previous work^24^, the needle prototype was made of six-wires 0.25 mm in diameter, using the same material and dimensions as the wires used for the W6–0.25, placed around a seventh-fixed wire and kept together at the tip with a ring. The needle was let self-propel through a gelatine phantom (4% wt) using a step-by-step motion. The experiments showed that, when actuating one wire at the time, the mean slip ratio was 0.21. In this work, we replaced the ring with a shrinking tube, to reduce the diameter at the tip, and we used different gelatine concentrations (5% wt and 10% wt). The mean slip ratio for W6–0.25 in all three gelatine phantoms was 0.2, if we do not consider the increase of the value at the start of the experiment and at the interface between the different gelatine stiffness degrees.

One of the most common consequences of needle insertion into soft tissue is tissue displacement^38,39^. The wasp-inspired motion mechanism presented in this work can reduce the tissue motion and deformation, as Leibinger *et al*. demonstrated in their work^27^. The wires are continuously pushing and pulling the material around them, applying a constant strain to the material. Any viscoelastic material, such as the gelatine phantom or the tissue, shows stress relaxation, meaning that the stress decreases over time when a constant strain is applied. In our additional experiments, the plastic foil deformed before needle puncturing. This effect reflects what would happen when a needle has to penetrate an organ. The tissue boundary of the organ will be deformed under the load of the needle before puncturing occurs^36^.

The experiments have been done using tissue-mimicking phantoms made with porcine gelatine powder. In literature, various material for phantoms have been presented, among which agarose-, gelatin- or PVA-based phantoms are the most commonly used^40,41^. The type of material used to create the phantom for the study is not relevant as long as the stiffness of the phantom is comparable with that of soft tissues. In our study, we chose to use the gelatin-based phantoms, which are easy to fabricate and allowed us to create phantoms of different stiffness degrees in a fast manner.

In general, a phantom provides a controlled environment, allowing for performing repeatable experiments. However, in order to get a better understanding of the functionality of the needle in medical applications, experiments with real tissues are needed. For this reason, we also performed a brief *ex-vivo* performance evaluation in porcine liver, kidney, and brain using the W6D0.25 needle prototype. The needle was able to self-propel through the different tissue types, although the slip was higher than in the gelatine phantom. We believe that the high slip values were caused by the presence of various hard structures into the real tissue, such as muscle fibres, and cavities of vessels or connective tissue, which interfered with the needle motion.

During the experiments, we noticed that the wires were sometimes getting entangled. The reason might be related to our connection mechanism: the wires were only connected at the tip with a 10 mm long shrinking tube, and there was no system that kept them in place along the body length. Therefore, the wires could change position during the experiment. In future prototypes, wire entanglement might be avoided by adding several shrinking tubes along the body of the needle, each connected to a different wire.

During percutaneous procedures, needles are visualized using X-ray or ultrasound. Despite the use of radiation, X-ray imaging is preferred over ultrasound, because of the ease of visualization of the needle in the image. To avoid insertion of multiple medical instruments and reduce the operation time, there is a need for needles that include visualization in real time and functionalities such as being able to take a sample or inject a drug. Our prototypes contain multiple wires, each of which can be easily replaced with an optical fibre to allow real-time visualization of the needle inside the body, and tubes to administer drugs or to perform biopsies.

Summarizing, in this study, we showed the advantage of using an ovipositor-inspired motion mechanism to prevent buckling of needles with high length-to-diameter ratio (>200) during deep insertion into tissue-mimicking phantoms. Additionally, we demonstrated that a thicker needle with more wires performs better than a thinner needle with fewer wires. Finally, we showed that the wires can be replaced with functional elements, such as optical fibres and tubes, to create a multifunctional needle without increasing the complexity of the design. The use of such a needle design and motion mechanism could improve the accuracy and safety during delicate medical procedures.

## Supplementary information

Supplementary Information

Supplementary Video S1

## Data Availability

The authors declare that all relevant data are available within the paper or in its Supplementary Material.
