# Supplementary material for: Experimental evaluation of a self-propelling bio-inspired needle in single- and multi-layered phantoms
Source: Sci Rep. 2019 Dec 27;9:19988. doi: 10.1038/s41598-019-56403-0 (PMC6934672; doi:10.1038/s41598-019-56403-0)
Supplement: Supplementary file 1 — Supplementary Information [file 41598_2019_56403_MOESM1_ESM.pdf]

Experimental evaluation of a self-propelling bio-inspired needle in single- and multi-layered phantoms

M. Scali[1,], P. Breedveld[1], and D. Dodou[1]*

[1]*Delft University of Technology, BioMechanical Engineering, Delft, 2628CD, The Netherlands.*
** m.scali@tudelft.nl*

This PDF file includes:

Supplementary Methods
Supplementary Figure S1, Figure S2, Figure S3, Figure S4, Figure S5
Supplementary Video S1 (caption)

Supplementary Methods – Measurements with rheometer

The rheological properties, which are related to the Young's modulus, of four different gelatine concentration in water were measured by means of a rheometer (Rheometer AR-G2) and parallel plates (diameter 25 mm) at 20°C. The concentration used were: 5% wt, 8% wt, 10% wt and 15% wt. Two PVA masks with ten "holes" (diameter = 25 mm, height = 5 mm) were made. The day before the experiment, a liquid mixture of gelatine and water for each concentration was made. The mixture was then poured into the holes of the mask placed in a Petri dish. Three samples of each concentration were made. The samples were stored into a fridge overnight. The mask were covered with plastic foil to avoid the evaporation of water.

All the samples were placed out of the fridge at the same time and the tests started after 30 min. The order followed was: 10% wt, 5% wt, 8% wt and 15% wt. First we performed a strain sweep (changing strain at constant velocity) on the gelatine sample. This is used to define the linear viscoelastic region of the sample. The strain range used was 0.01 % to 10% at 10 rad/s. Then, right after that (approx. 5 min) a frequency sweep (changing angular frequency at a constant strain) with the same sample (approx. 12 min). This test was performed with the same sample. Within the linear strain regime we choose 0.5 % strain. The frequency range was 100Hz – 0.1 Hz. After that the gelatine sample with another concentration was placed on the plate and the tests were repeated (strain sweep and frequency sweep). The same procedure was repeated for the four concentrations. The replacement of the sample took in average 5 min. To ensure contact between the plates and the sample, an initial normal force was applied (10 %: 1.2 N, 5%: 0.25 N, 8%: 0.9 N, 15%: 1 N).

A total of three measurements for each concentration was performed. The strain sweep was performed only for the first experiment. After that, the linear strain range of the four concentration was known and there was no need to repeat the tests for the other samples. The second measurement started approx. 2h after the beginning of the experiment. The third measurement started approx. 4h after the beginning of the experiment. The gap of the sample was approximately 5 mm for all the samples.

From these tests, we obtained values for the storage modulus (G') and the loss modulus (G''). The moduli were averaged across frequencies to calculate the dynamic shear modulus ($G*$). We calculated the absolute value of $G*$ as $G = \sqrt{G'^2 + G''^2}$. The elastic modulus E is related to G with the formula $E = 2G(1+\nu)$ where ν is the Poisson's ratio (for $\nu = 0.5$, $E = 3G$).

Fig. 1 shows the results of the measurements. The sample of 5% wt, 8% wt, 10% wt and 15% wt corresponded to elastic modulus of 5.3 KPa, 12 KPa, 17 KPa and 31 KPa, respectively.

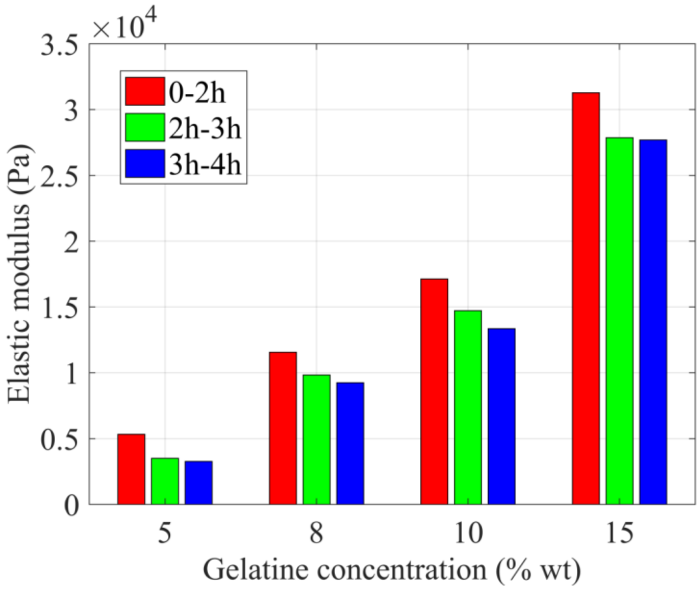

Figure 1. Elastic modulus (Pa) per gelatine concentration (% wt).

Supplementary Figure S1

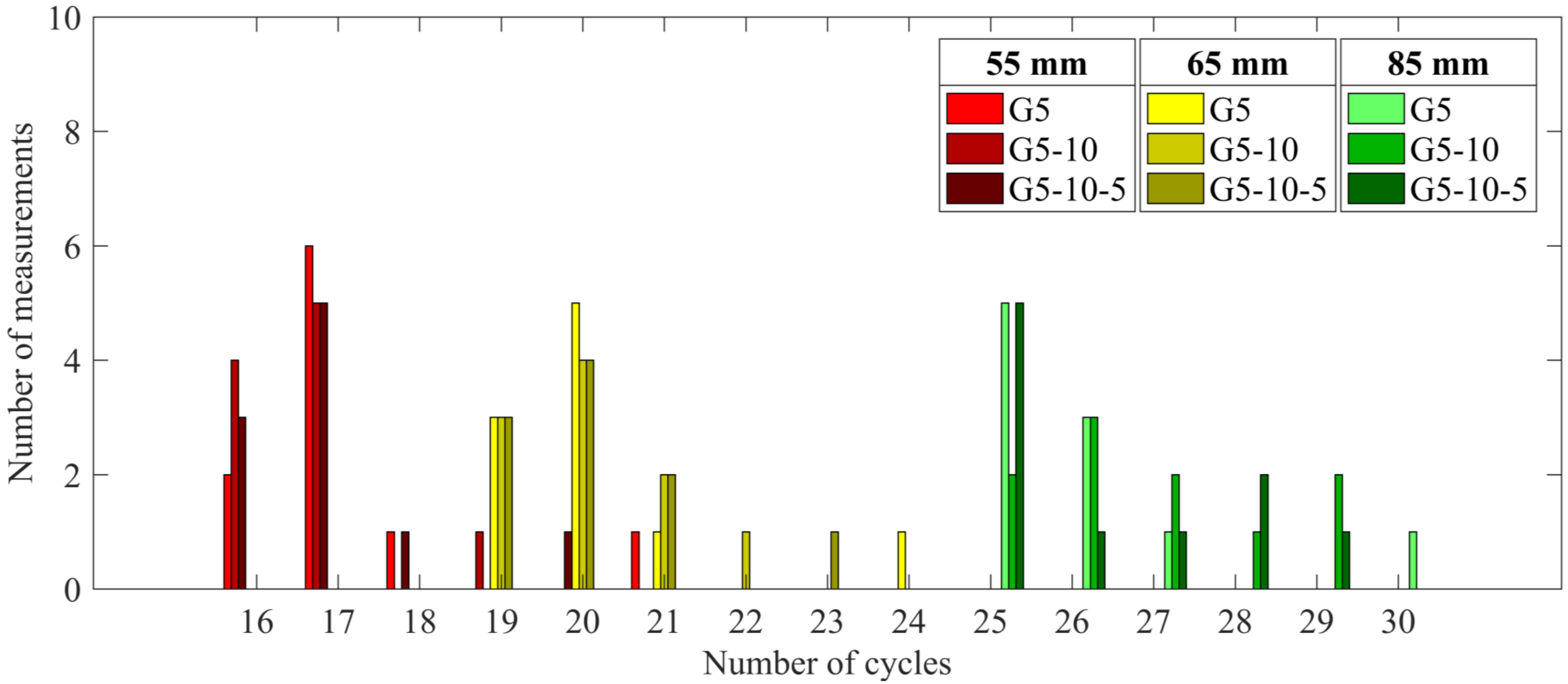

Figure S1. Experiment with W6-D0.25 prototype actuated with step-by-step motion. Number of measurements per gelatine sample that reached a reference depth in the same number of cycles.

Supplementary Figure S2

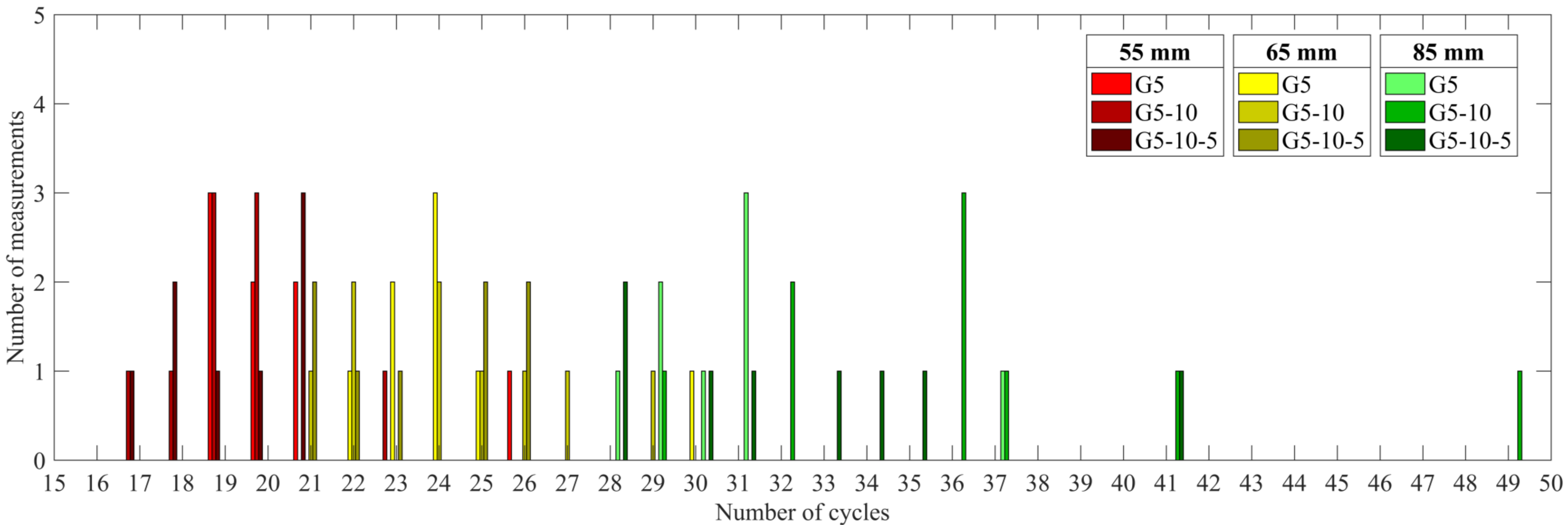

Figure S2. Experiment with W6-D0.125 prototype actuated with step-by-step motion. Number of measurement per gelatine sample that reached a reference depth in the same number of cycles.

Supplementary Figure S3

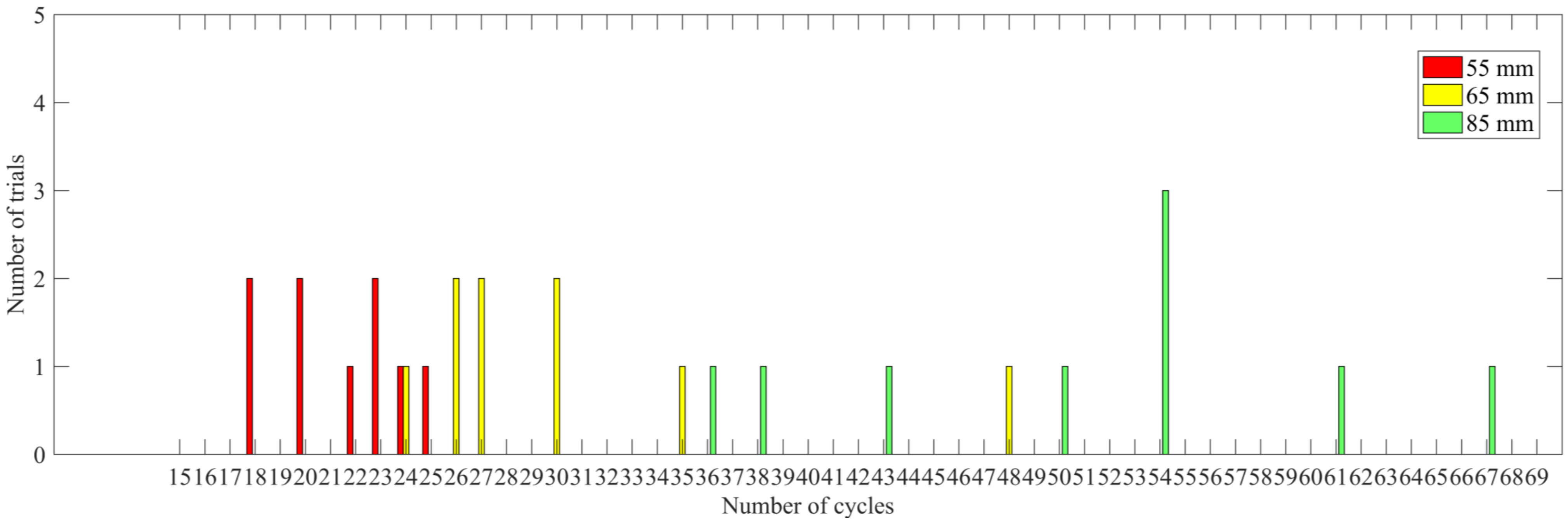

Figure S3. Experiment with W3-D0.25 prototype actuated with step-by-step motion. Number of measurement per gelatine sample that reached a reference depth in the same number of cycles.

Supplementary Figure S4

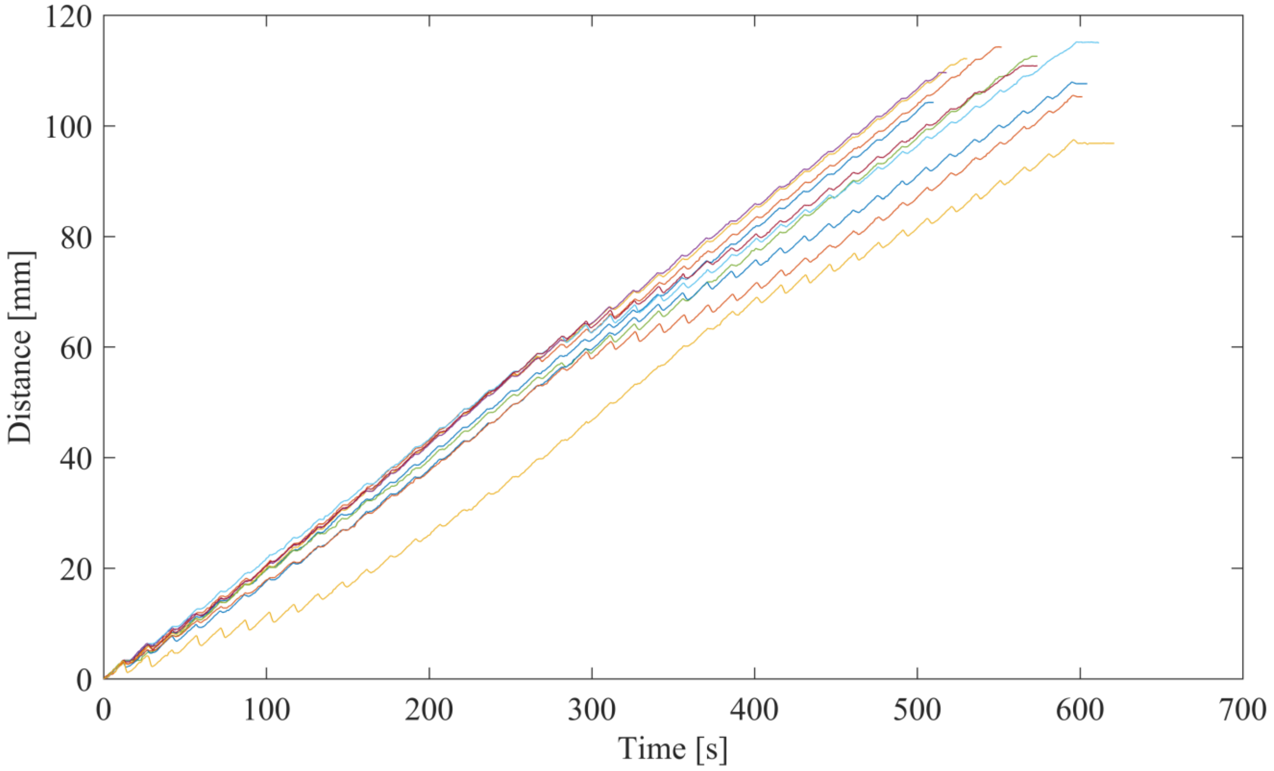

Figure S4. Distance travelled by the needle inside the gelatine phantom during the experiment with continuous motion.

Supplementary Figure S5

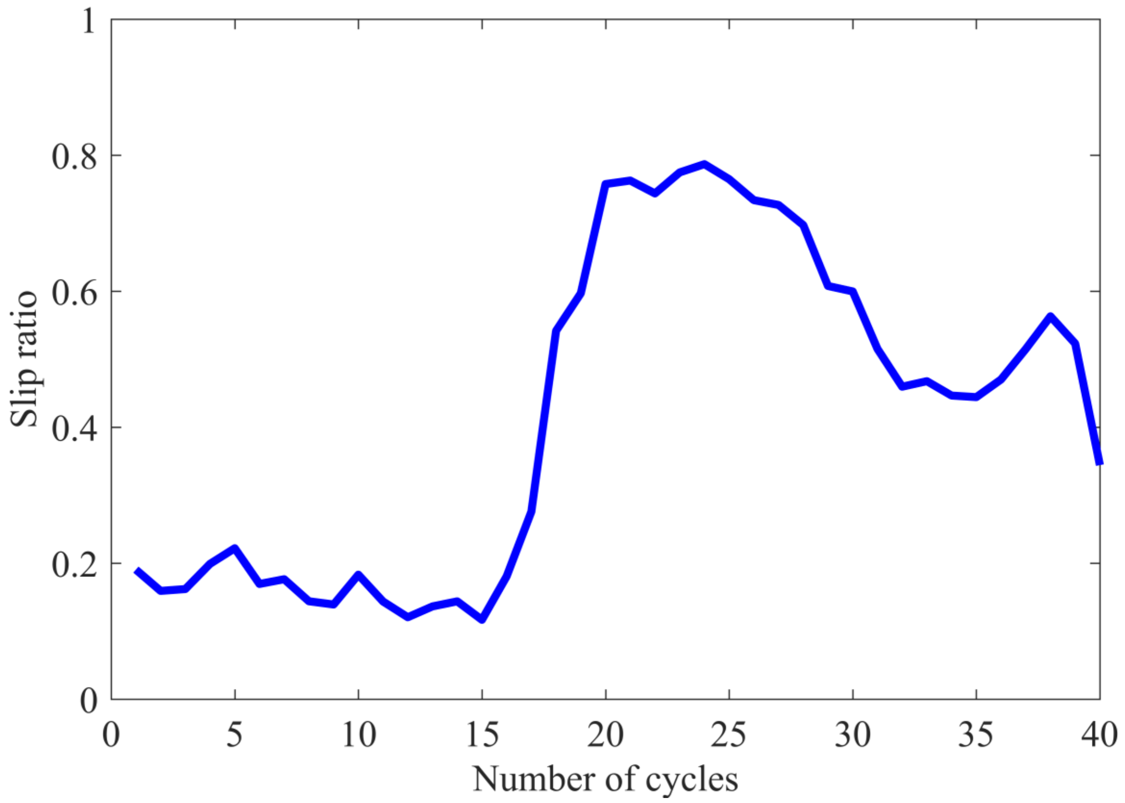

Figure S5. Slip ratio during the insertion of the W6-D0.25 prototype inside 5%-15%.

Supplementary Video S1. Video of the needle puncturing a plastic foil placed between two layers of gelatine 5% wt (speed 2x).
